# Thymic cancer: A not-so-indolent cause of pericardial effusion

**DOI:** 10.1016/j.amsu.2021.102866

**Published:** 2021-09-16

**Authors:** Adrian Whiting, Jonathan Vincent M. Reyes, Saad Ahmad, Mark N. Sayegh, Talal Almas, David Song

**Affiliations:** a NYU Langone Hospital - Long Island, Department of Medicine, Mineola, NY, USA; b Icahn School of Medicine at Mount Sinai, Elmhurst Hospital Center, Department of Medicine, Elmhurst, NY, USA; c Royal College of Surgeons in Ireland, Dublin, Ireland; d St. John's Riverside Hospital, Department of Medicine, Yonkers, NY, USA

**Keywords:** Pericardial effusion, Thymic cancer, Keratinizing squamous cell carcinoma

## Abstract

The incidence of pericardial effusion in the U.S. is roughly 3.4% [1]. While most causes of pericardial effusions are indolent and transient, malignancy is a much more insidious cause that cannot be overlooked. Most cases of documented pericardial effusion secondary to malignancy have been due to mass effect from benign thymic tumors, such as thymomas. Our case highlights a 41-year-old male who presented with a dry cough and epigastric pain, found to have a large pericardial effusion and incidental thymic mass. The mass was biopsied and found to be keratinizing squamous cell carcinoma. This case expands our knowledge base as clinicians that pericardial effusions can be caused by malignant extension of tumors, rather than simply by mass effect of benign tumors.

## Highlights

• Thymic carcinoma can extend into the pericardium, although classically it extends to the lungs.
• Although rare, metastatic disease should remain on the differential for pericardial effusions.
• Platinum based chemotherapy such as Cisplatin remains the standard of care for thymic malignancies that are locally advanced as well as metastatic disease.

## 1 Introduction

Pericardial effusions can range from small, benign, and chronic to large and acute cases requiring urgent medical attention. The general prevalence of pericardial effusions in the United States has been shown to be roughly 3.4% in a post-mortem study of patients who succumbed to non-cardiac illnesses [1]. Pericardial effusions are fluid accumulations in the pericardial sac, transudate, exudate, or frank blood, greater than the physiologic pericardial fluid volume (15–50 mL), which can become clinically symptomatic and even lead to cardiac tamponade if the volume exceeds 100–150 mL [2]. The causes of pericardial effusions range from infectious, inflammatory/rheumatologic, traumatic, idiopathic, and most importantly, neoplastic [2].

While the prevalence of idiopathic asymptomatic pericardial effusions in the general population is low, the prevalence of pericardial effusion secondary to neoplasm can reach up to 23% [2]. Pericardial effusions may be caused by both primary pericardial malignancies, such as mesothelioma, and metastatic cancers such as breast and lung cancers, Hodgkin lymphoma, and sarcomas [3,4]. Pericardial effusion secondary to malignancy has been hypothesized to arise due to four different mechanisms: (1) direct extension or metastatic spread via blood or lymphatics, (2) chemotherapy toxicity, (3) radiation toxicity, and (4) opportunistic infections in the setting of immunosuppressive drug therapy [5].

As demonstrated by the patient in our case, pericardial effusion secondary to thymic cancer is rare. Thymic carcinoma arises from the epithelial cells of the thymus gland. Thymic carcinoma is responsible for approximately 1% of all thymus cancers. Unfortunately, it usually manifests in the late stage and has a 5-year survival rate between 30 and 50%. The majority of thymic carcinomas manifest as a cough, chest pain, phrenic nerve palsy, and superior vena cava syndrome [6]. Although a rare malignancy, thymoma comprises 20% of all mediastinal neoplasms and up to 50% of all the anterior mediastinal tumors. Other malignancies found in this region include thymic carcinoma, lymphoma, and teratoma [7]. Thymic epithelial tumors are separated into two categories: thymomas and thymic carcinomas. The thymus is the main site of maturation for T Cells and is known to be integral to our adaptive immune response. A vast array of autoimmune disorders such as Myasthenia gravis, pure red cell aplasia, and hypogammaglobulinemia have been known to be associated with thymomas; meanwhile, patients with thymic carcinoma seldomly develop any associated autoimmune pathology [8]. In addition, this work has been reported in accordance with SCARE [9].

## 2 Case presentation

Our patient is a 41-year-old man with a past medical history of obesity, fatty liver disease, hypertension, former cigarette smoker, and prior coronavirus disease 2019 (COVID-19) infection in April 2020 who presented to the emergency department complaining of a one-month history of dry cough and a one-week history of epigastric pain and fatigue. The patient denied having any fever, rhinorrhea, sore throat, dysphagia or odynophagia, hematochezia, changes in his bowel habits, or unintentional weight loss. The patient endorsed nausea with early satiety and shortness of breath with exertion and orthopnea progressively worsening two-weeks before presentation. The patient was never hospitalized, intubated or received any treatment for COVID-19.

**Fig. 1:**
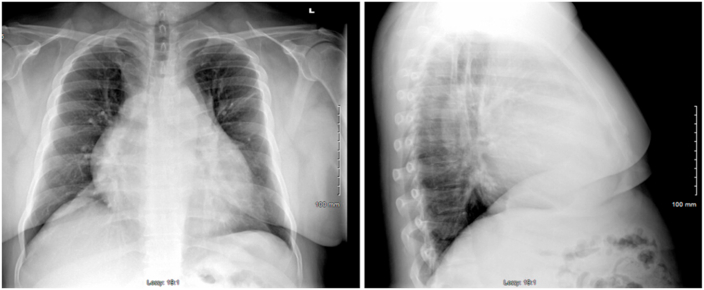
Frontal and lateral view demonstrating enlarged cardiac contour with obscuration of the pulmonarty arteries; suspicious pericardial effusion.

**Fig. 2:**
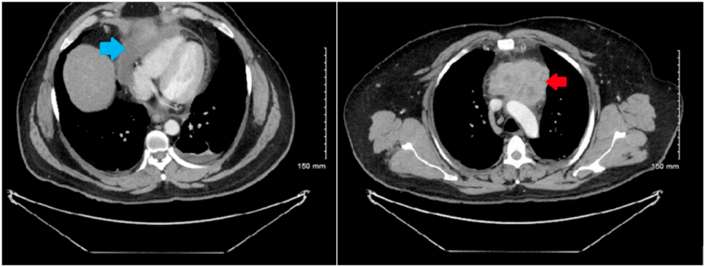
CT Chest with contrast demonstrating a heart that is normal in size with a right pericardial effusion measuring 98 × 29 mm (blue arrow). Large irregular inhomogeneous retrosternal/superior mass measuring 7.8 × 5.7 × 5.8 cm (red arrow). (For interpretation of the references to colour in this figure legend, the reader is referred to the Web version of this article.)

The patient's physical exam was significant for a non-productive cough and bilateral abdominal tenderness to palpation. There were no distant heart sounds and jugular vein distension. Lab testing in the emergency department revealed an elevated white blood cell count to 11.78 with eosinophilia to 5.4. Lipase was elevated to 71 and the patient was slightly hyponatremic with a sodium of 133. Other electrolytes were unremarkable. The patient's troponin and brain natriuretic peptide were within normal limits. A Quantiferon Gold test was positive, chest radiography was consistent with an enlarged cardiac contour consistent with a pericardial effusion **(**Fig. 1**)**. Bedside cardiac ultrasound confirmed the presence of a large pericardial effusion without physiologic tamponade. Chest computed tomography (CT) with contrast was performed which revealed a 7.8 cm × 5.7 cm x 5.8 cm irregular, inhomogeneous retrosternal/superior mediastinal mass **(**Fig. 2**)**.

The patient was admitted to the cardiac care unit where a pericardiocentesis was performed and 1000-mL of bloody fluid was removed and sent for culture. All fungal and acid-fast cultures and stains came back negative. Although a positive quantiferon gold test, with negative acid-fast cultures, tuberculosis (TB) treatment was not initiated given low suspicion for TB. Interventional Radiology (IR) was consulted and IR-guided biopsy of the mass was performed and demonstrated invasive keratinizing squamous cell carcinoma of thymic origin. An immunohistochemical study was performed and the neoplastic cells were positive for CD117 and CD5. This immunostaining profile was in favor of a thymic origin squamous cell carcinoma. CD117 positive cells are found to be present in roughly 84% of cancers of thymic origin. However, CD117 is also present in about 27% of poorly differentiated lung carcinoma [10]. Lack of CD4 or single CD8 or CD20 positive cells is indicative of a malignant thymic cancer as opposed to a benign epithelial thymic tumor [11]. The cells in our patient's tumor lacked CD4. In addition, this tumor histologically shows lobulated growth with hyaline stroma, also in favor of a thymic origin. However, it is important to note that both clinical and radiologic correlations are more important for the final determination of its origin.

After resolution of the patient's acute symptoms due to pericardial effusion, the patient was followed up in the outpatient setting by the cardiothoracic surgery team and the oncology team. The team decided to treat his differentiated thymic cancer as a non-small cell lung cancer and the patient was medically treated with concomitant radiation and chemotherapy, including Cisplatin and Etoposide, followed by Pemetrexed and Carboplatin. The patient continued to receive treatment and followed up regularly with no recurrence of the pericardial effusion.

## 3 Discussion

While pericardial effusions are generally uncommon, when they arise, they are usually asymptomatic and due to benign causes. The patient in our case demonstrated a rare cause of pericardial effusion: malignant extension from a thymic tumor. This case report illustrated an important clinical scenario to be aware of when assessing pericardial effusions in the setting of a mediastinal mass. Recent and past literature have reported benign thymic tumors causing pericardial effusions through mass effect, as demonstrated by Nishi T et al. [12], as well as massive mediastinal tumors mimicking pericardial effusions, as demonstrated by Bernstein A et al. [13]. However, to our knowledge, no literature has demonstrated a pericardial effusion secondary to malignant extension from a mediastinal/thymic tumor.

The natural course of thymic carcinoma is to first locally metastasize to the surrounding lymph nodes in the cervical region. Systemic metastasis in a majority of cases is to the lungs [14], less likely to the heart or pericardium. Cancers that are most likely to cause a malignant pericardial effusion are leukemias, lymphomas, melanoma, lung and breast cancers [14]. Once identified as a malignant pericardial effusion, the treatment of choice is surgery, carrying a higher mortality than a simple pericardiocentesis, but with better long-term outcomes [15]. An important part of the clinical work up of a pericardial effusion with a mediastinal mass is to consider all possible differentials. One important differential that fits this scenario is myasthenia gravis, which can present with a thymoma in the anterior mediastinum and is usually a benign tumor.

In terms of thymic malignancies, surgery remains the treatment of choice for operable thymic tumors. Platinum based chemotherapy such as Cisplatin remains the standard of care for thymic malignancies that are locally advanced as well as metastatic disease. Histone deacetylase inhibitors have demonstrated some response in thymoma; sunitinib, a receptor tyrosine kinase inhibitor, may be active in thymic carcinoma [8]. Thymic malignancies may be staged based on the Masaoka staging system. This system focuses on the integrity of the thymic capsule, the appearance of macroscopic or microscopic invasion into nearby structures, and metastatic spread [16].

## 4 Conclusion

This case adds evidence to our clinical repertoire that pericardial effusions can have insidious malignant causes. While some of the more extreme causes of pericardial effusions such as TB and hypothyroidism should always be considered given a patient's demographics and risk factors, metastatic disease should remain on the differential until proven otherwise. Our patient had a very strong geographic risk factor for TB-induced pericardial effusion with minimal social risk factors, such as smoking or tobacco use, grounding in a particular diagnosis in this case caused us to overlook this potential differential diagnosis and delay treatment. Thus, it is important to approach each case of pericardial effusion with an open mind, avoid diagnosis grounding and fully evaluate each piece of clinical information pertinent to the patient.

## Provenance and peer review

Not commissioned, externally peer-reviewed.

Written informed consent was obtained from the patient for publication of this case report and accompanying images. A copy of the written consent is available for review by the Editor-in-Chief of this journal on request.

## Please state any conflicts of interest

None.

## Please state any sources of funding for your research

None.

## Ethical approval

Obtained.

## Consent

Obtained.

## Author statement

AW, JVR wrote the abstract, introduction, case, discussion, conclusion.

SA, DS, TA, MNS performed critical edits and final revision, figures.

## Registration of research studies

1. Name of the registry: NA
2. Unique Identifying number or registration ID: NA
3. Hyperlink to your specific registration (must be publicly accessible and will be checked): NA

## Guarantor

Talal Almas.

RCSI University of Medicine and Health Sciences.

123 St. Stephen's Green Dublin 2, Ireland.

Talalamas.almas@gmail.com.

+353834212442.

## Declaration of interest

None.

## Disclosure

None.

## Declaration of competing interest

None.
